# Biomineralisation to Increase Earth Infrastructure Resilience

**DOI:** 10.3390/ma15072490

**Published:** 2022-03-28

**Authors:** Ana Bras, Hazha Mohammed, Abbie Romano, Ismini Nakouti

**Affiliations:** 1 Built Environment and Sustainable Technologies (BEST) Research Institute, School of Civil Engineering and Built Environment, Liverpool John Moores University, Liverpool L3 3AF, UK; h.b.mohammed@2018.ljmu.ac.uk (H.M.); abbieromano@googlemail.com (A.R.); 2 Centre for Natural Products Discovery (CNPD), School of Pharmacy and Biomolecular Sciences, Liverpool John Moores University, Liverpool L3 3AF, UK; i.nakouti@ljmu.ac.uk

**Keywords:** biological methods, earth, cement, self-healing, infrastructure

## Abstract

The vulnerability of buildings and structures to rain and flooding due to a lack of adaptive capacity is an issue all over the world. Exploring the bio-resources availability and engineering performance is crucial to increase infrastructure’s resilience. The current study analyses earth-based mortars using mineral precipitation as a biostabiliser (bio) and compares their performance with cement-based mortars. Cultures of *S. oneidensis* with a concentration of 2.3 × 10^8^ cfu/mL were used to prepare earth-based and cement-based mortars with a ratio of 6% of binder. Microstructure analyses through SEM/EDS, water absorption, moisture buffering, mechanical strength, and porosity are discussed. The biostabiliser decreases water absorption in tidal-splash and saturated environments for earth and cement mortars due to calcium carbonate precipitation. The biostabiliser can prevent water migration more effectively for the cement-based (60% reduction) than for the earth-based mortars (up to 10% reduction) in the first 1 h of contact with water. In an adsorption/desorption environment, the conditions favour desorption in cem+bio, and it seems that the biostabiliser precipitation facilitates the release of the chemicals into the mobile phase. The precipitation in the earth+bio mortar porous media conditions favours the adsorption of water molecules, making the molecule adhere to the stationary phase and be separated from the other sample chemicals. The SEM/EDS performed for the mortars confirms the calcium carbonate precipitation and shows that there is a decrease in the quantity of Si and K if the biostabiliser is used in cement and earth-mortars. This decrease, associated with the ability of *S. oneidensis* to leach silica, is more impressive for earth+bio, which might be associated with a dissolution of silicate structures due to the presence of more water. For the tested earth-based mortars, there was an increase of 10% for compressive and flexural strength if the biostabiliser was added. For the cement-based mortars, the strength increase was almost double that of the plain one due to the clay surface negative charge in the earth-based compositions.

## 1. Introduction

The vulnerability of buildings and structures to rain and flooding due to a lack of adaptive capacity is an issue all over the world according to the United Nations Development Programme (UNDP). Indeed, 300 million new houses are needed in the world by 2030 to provide accommodations for three billion people. Just in South Asia and West Africa, there is an enormous demand for housing that needs to be catered for within a short span of time. Half of the population in the world lives in rural areas, where earth-based construction is predominant [1,2,3]. However, the local earth-based materials for construction cannot withstand inundation, leading to housing collapse and affecting livelihoods. Understanding and exploring the bio-resources availability and their engineering performance is crucial to increase housing resilience for flooding with low carbon impact.

By adding different admixtures and additives to the soil, there is a modification of its characteristics. This stabilisation technique is commonly adopted in earth-based houses but can generate concerns in the soil environment. The addition of such cementitious admixtures to the soil as lime or cement can improve the soil strength but change the environment in a permanent way and requires the emission of greenhouse gases during admixtures manufacturing [4,5,6,7,8,9,10,11,12,13,14]. This highlights the need for a different and sustainable stabilisation technique for soils, such as the use of biological methods. The biological methods include techniques such as mineral precipitation and biopolymer and biofilm accumulation [1,4,5,6].

The mineral precipitation consists of precipitation of calcium to fill the pores of the soil, enabling to increase soil bonding. Calcium carbonate precipitation via bacterial activity has gained popularity as a soil improvement technology in recent years. It is defined as a novel and environmentally friendly procedure [15,16,17]. This novel technology has benefits over traditional chemical treatments, which could be toxic and damaging to the environment, as well as having a short injection distance. In comparison to chemical treatments, the biological approach is cost-effective [18,19]. Several literature studies have found that using microbial-induced calcite precipitation to increase bonding, shear strength, and decrease permeability in sandy and gravelly soils is quite efficient [19,20]. There is currently a limited characterisation of the materials that directly compare soil/earth-based infrastructure and self-healing behaviour based on biomineralisation [21,22,23,24,25,26]. The work presented in Ref. [21] aimed to test four different bio-based materials to create an earth mortar using bio-consolidation mechanisms to extend service life and boost durability for potential implementation as a repair mechanism for earth construction. It was detected that the use of the bio-product increased the sensitivity of earth-based construction to moisture buffering cycles, contributing to less mass loss during sorption cycles and increasing the long-term performance of these earth-based composites. Furthermore, a research study conducted by Chou et al. [27] reported that the microbial activity contributed to the blocking of the porous medium of the soil. Biofilms are natural and abundant in natural environments. Whereas biofilms protect microorganisms from chemical, physical, and biological external stimuli [28], the formation of biofilms in soils has a significant impact on the stability of soil bonding, causing the breaking down/dissolving of minerals, organic carbon degradation and sequestration, and reduction in hydraulic conductivity [29].

Biopolymers are natural polymers with high tensile strength, and they are harmless and biodegradable. Biopolymer use for soil improvement has grown in popularity in recent years [16,29]. They are recognised for their gel-like extracellular polymeric substance. This method relates to the use of biopolymers as a chemical cementing agent in soils. Gellan gum, xanthan gum, gum from algae, bacteria-produced beta-glucan, chitosan from shellfish, casein from dairy agar products and guar gum, and leguminous plants from plants are all examples of biopolymers [30]. In order to obtain the desired engineering performance, the extracted biopolymers from diverse sources are purified and dried, then combined with water and soils at a predetermined ratio. The biopolymer treatment of soil is used to increase the liquid limit and improve the strength of soils [31], change water repellence, enhance erosion resistance, and reduce hydraulic conductivity [32].

Bio-based soil stabilisation technologies and biopolymers treatment have been studied extensively to determine the way they affect certain engineering characteristics; they are at the vanguard of field-scale applications. Due to the variables of soil physical properties and testing, appropriate experimental data have yet to be accumulated and analysed, which has hampered further progress in developing unique yet effective soil development approaches.

The knowledge and demonstration of modern and durable earthen material with bio-based techniques for housing in different contexts can act as a catalytic agent to improve earth and tailor enhanced solutions for housing. Improving the resilience of local housing directly contributes to the UN Sustainable Development Goal (SDG) 11, to mitigate significant social and economic damage associated with flooding.

The significant cost of traditional stabilised earthen material can be reduced by dropping cement/hydraulic lime use in favour of bio-based alternatives. This research intends to find new opportunities to increase infrastructure resilience through self-recovery/adaptive solutions from the re-use of bio-resources. This contributes to UN SDG 9 as infrastructure and innovation investments are critical for economic growth and development, providing new jobs and promoting energy efficiency. Global housing requirements pose a significant financial and environmental challenge under the climate emergency.

This study focuses on the potential of using mineral precipitation as stabilisers in earthen construction materials. By presenting a low embodied energy and carbon footprint, these biostabilisers have some advantages over cement, and they are widely available around the world, providing suitable mechanical properties, durability, and improved hygroscopic performance. Earth-based mortars’ performance with the biostabiliser and without it are compared with cement-based mortars with or without the biostabiliser. Cultures of *S. oneidensis* with a concentration of 2.3 × 10^8^ cfu/mL are used. The biostabiliser is then used to prepare earth-based and cement-based mortars with a ratio of 6% of binder. The study is conducted at different levels of relative humidity (RH) as, at a low RH, capillary suction is more efficient, whereas, in wet/humid porous media, diffusion coefficients decrease for gases but play a more important role for the mobility of ions, which is greatest in completely water-filled pores. The microstructure analysis through SEM/EDS, water absorption via capillary, moisture buffering, mechanical strength, and porosity results are analysed.

## 2. Materials and Methods

### 2.1. Soil

**Figure 1:**
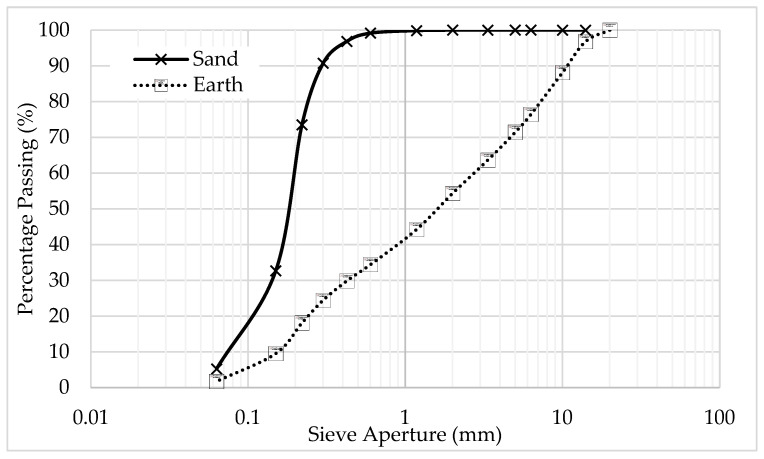
Grain size distribution of earth and sand.

The sand and earth grain size distribution is presented in Figure 1. They were characterised in accordance with EN 1015-1. The dry bulk density of sand is 2500 kg/m^3^ and of earth is 2527 kg/m^3^.

### 2.2. Binders

#### 2.2.1. Cement

Type one ordinary Portland cement (CEMI) 52.5 N was used to produce the cement-based mortars in accordance with BE EN 197-1. The specific gravity is 3.13 g/cm^3^.

#### 2.2.2. Lime

Natural hydraulic lime 3.5 was used for the mix design of earth-based mortars, which was supplied from a national builder’s merchant—where the requirements are defined within BS EN 459-1, 2015. The lime dry bulk density is 2700 kg/m^3^.

### 2.3. Mix Design

A flow table test was used to ensure that each mix design had a spread value between 155 ± 5 mm, according to BS EN 1015-3. This enabled the calibration of the consistence of each sample. Four types of mortars were developed: earth-based with and without biostabiliser (bioproduct) and cement-based with and without bioproduct (Table 1).

**Figure 2:**
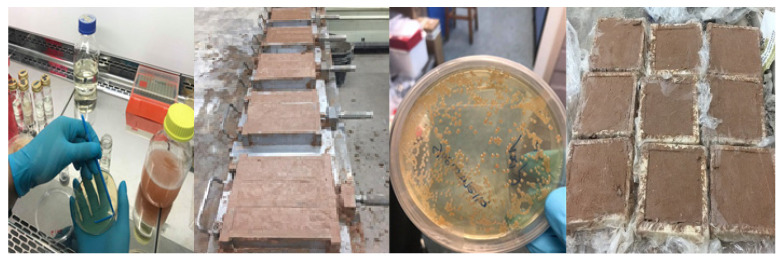
Bioproduct and mortar prismatic and square samples. The square samples are for moisture buffering test.

Cultures of *S. oneidensis* were incubated at 30 °C to achieve the concentration of 2.3 × 10^8^ cfu/mL. The bioproduct was then used to prepare earth-based and cement-based mortars with a ratio of 6% of binder. Figure 2 presents the bioproduct and mortar samples.

### 2.4. Methods for Testing

The earth-based and cement-based mortars were tested according to the following procedures (Table 2), using the equipments available at Liverpool John Moores University.

## 3. Results and Discussion

**Figure 3:**
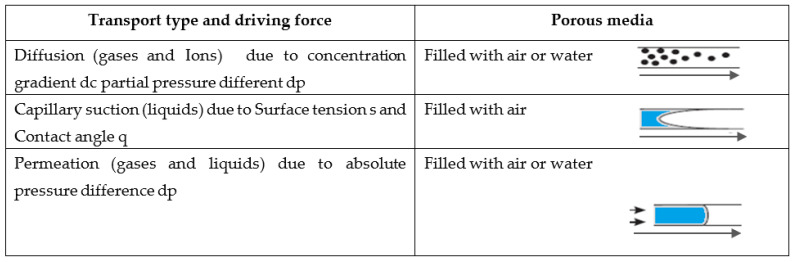
The different types of transport processes in an earth or cement-based medium based on Ref. [33].

The porous medium of each mortar, which includes cracks and voids within earth- and cement-based materials, enables the transport of carbon dioxide and oxygen and water and ions, with a direct impact on the durability of the structures. The transport is caused by a change in the concentration of the ions, pressure gradients for gases and water, differences in the absolute pressure for gases and water, migration, and capillary forces. Figure 3 presents the different types of transport processes in an earth- or cement-based medium.

In order to understand the influence the biostabilisers have in the micro and macro properties of the cement- and earth-based mortars, this chapter focuses on the discussion of the results regarding the microstructure modifications detected through SEM/EDS, water absorption via capillary, moisture buffering, mechanical strength, and porosity.

### 3.1. Bulk Density

**Figure 4:**
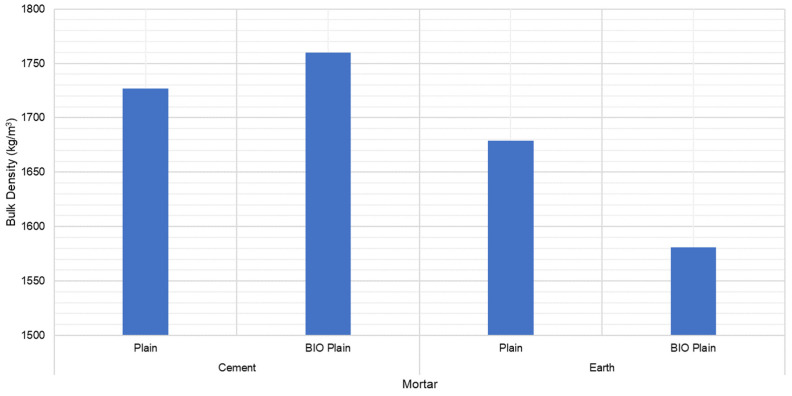
Bulk density for the cement- and earth-based mortars (plain) with and without biostabiliser (bio). Coefficient of variation (CoV) per family of mortar: cement mortar: 1.0%; cem+bio: 0.5%; earth mortar: 4.8%; earth+bio: 1.0%.

The following figure (Figure 4) presents the bulk density for the cement- and earth-based mortars (plain) with and without biostabiliser (bio).

The results show that the impact of the biostabiliser on the density slightly change the density by less than 6% for cement and earth-based mortars.

### 3.2. Water Absorption via Capillary

Capillary suction corresponds to the transport of water in mortar porous media due to surface tension acting in the capillaries. It is a function of the pore structure (including the tortuosity and continuity of the capillaries and their radius) of the mortar. Capillary tests were done using EN 1015-18. The samples were placed in an oven at 30 °C until the mass change was less than 0.1%. The weights of the samples were registered at 0′, 5′, 15′, 30′, 1 h, 2 h, 3 h, and 21 h up to when the water absorption stabilised and reached an asymptotic value.

**Figure 5:**
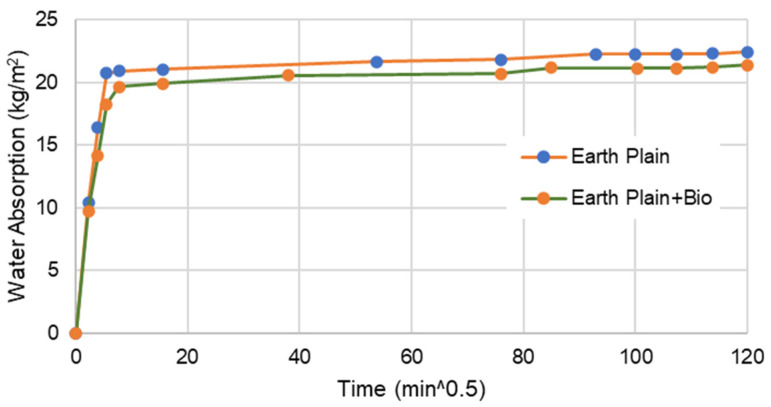
Water absorption for bio and non-bio earth mortar samples after 28 days. Coefficient of variation (CoV) per family of mortar: earth mortar: 2.8%; earth+bio: 3.8%.

**Figure 6:**
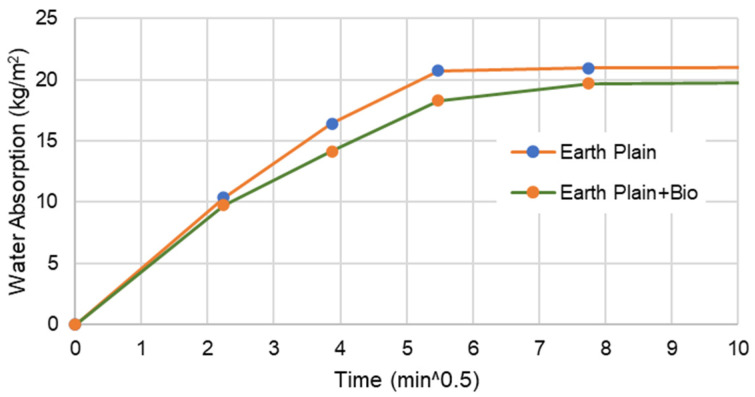
Water absorption for bio and non-bio earth mortar samples during the first 1 h.

**Figure 7:**
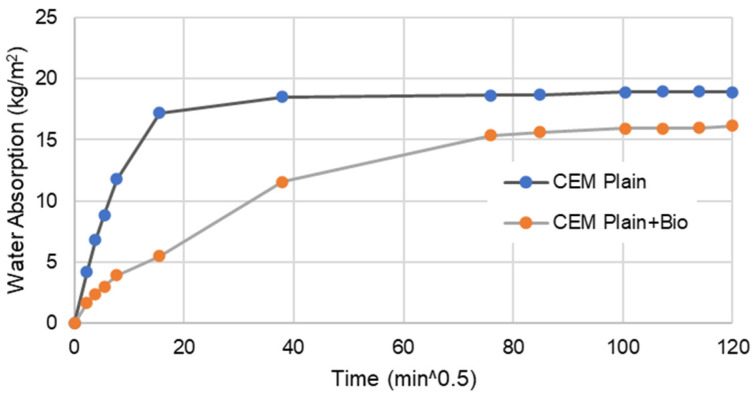
Water absorption for bio and non-bio cement mortar samples after 28 days. Coefficient of variation (CoV) per family of mortar: cement mortar: 9.1%; cem+bio: 5.5%.

**Figure 8:**
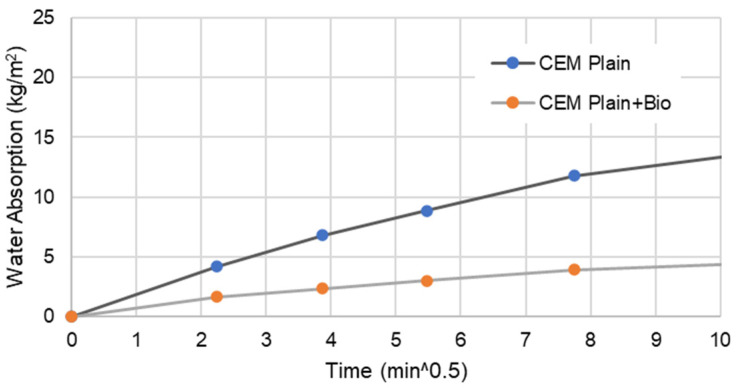
Water absorption for bio and non-bio cement mortar samples during the first 1 h.

Figure 5, Figure 6, Figure 7 and Figure 8 present the capillary water absorption for the earth- and cement-based mortars.

It is observed that the initial water absorption tends to be lower for the bio samples than the non-bio samples, which is a desirable result. At this range, the water absorption via the capillary tends to reduce by at least 60% if the mineral precipitation is promoted in cement-based mortars. For earth mortars, the contribution of this mineral precipitation is between a 5 and 10% reduction in the initial stage of water absorption. This emphasises that the biostabiliser can prevent water migration more effectively for cement-based than for earth-based mortars in the initial stage of the capillary absorption. 

In the asymptotic value stage—when the samples tend to become saturated—the biostabiliser shows a constant contribution of a 5% decrease in water absorption for the earth-based mortars. For the cement+bio mortars, the contribution to decrease absorption was above 15%.

In a flooding situation, there is always a stage when there is water absorption in a non-saturated medium and in a saturated medium. The first corresponds to the beginning of the capillary curve (Figure 6 and Figure 8), when a structure or a building is exposed to the tidal-splash water. The second corresponds to structures of the building under water, when the medium (earth or cement) is already saturated; in a capillary curve, this corresponds to the plateau. Therefore, it seems that this biostabiliser presents higher benefits to minimise the migration of water-soluble ions in the tidal-splash zones of earthen constructions than in underwater environments.

Since very impressive results are obtained during the first 1 h of testing, where the water is still close to the mortar surface, from a mineral precipitation point of view, it seems that the capillary action was impeded by the microstructure formed near the surface by *S. oneidensis* in a more impressive way for the cement than for the earth-based mortars, which explains the improved results for the cement-based compared to the earth-based mortars. The long-term analysis shows, however, that the microbial precipitation effect is detected deeply in the mortars through the differences between the asymptotic value of the samples with or without bio. This is confirmed through the SEM/EDS results discussed below.

### 3.3. SEM/EDS

**Figure 9:**
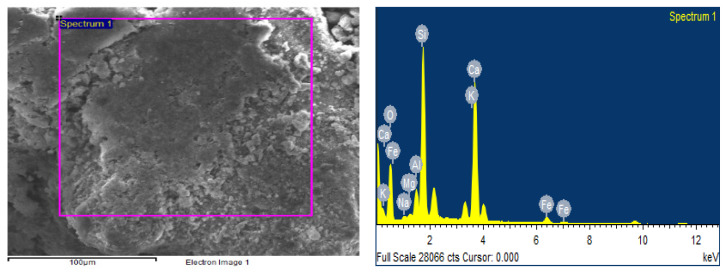
EDS of cement plain mortar.

**Figure 10:**
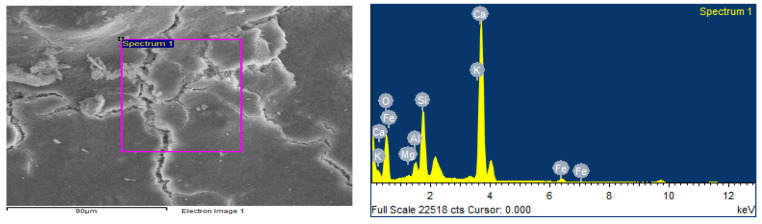
EDS of cem+bio mortar.

**Figure 11:**
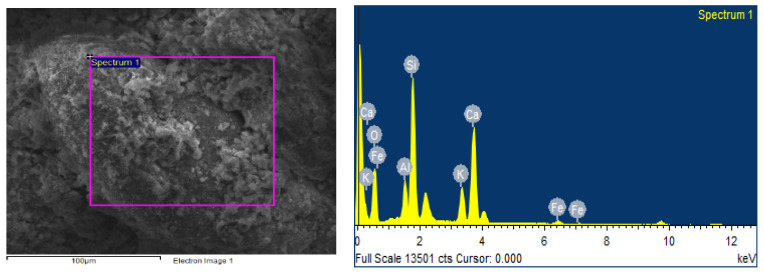
EDS of earth-based plain mortar.

**Figure 12:**
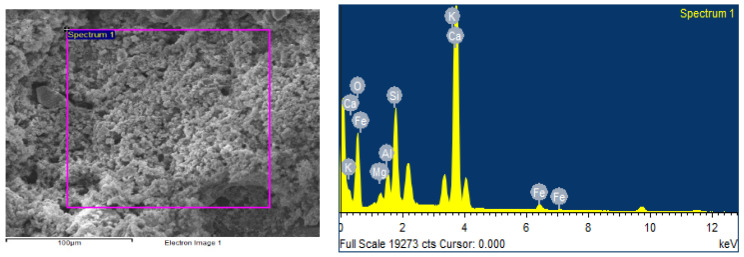
EDS of earth+bio mortar.

Each mortar sample was crushed, and the inner part was coated in gold in order to investigate the bonding characteristics between the cement paste and the aggregates with or without the biostabiliser. In order to confirm the formation of calcium carbonate precipitation, an EDS analysis was performed for the cement-based (Figure 9 and Figure 10 and Table 3) and earth-based (Figure 11 and Figure 12 and Table 4) mortars.

The measurement in different areas was focused, and the corresponding peaks are shown in Figure 9 and Figure 10. The CaCO_3_ is associated with an increased quantity of Ca and oxygen, as observed in the spectrum of the cem+bio in comparison to the cement plain. The presence of Si is associated with the hydration of cement paste and the presence of sand. The details of the EDS are presented in Table 3.

In order to confirm the formation of calcium carbonate precipitation in the earth-based mortars with the biostabiliser, an EDS analysis was performed (Figure 11 and Figure 12 and Table 4). The measurement in different areas was focused, and the corresponding peaks are shown in Figure 11 and Figure 12. The CaCO_3_ is associated with an increased quantity of Ca, as observed in the spectrum of the earth+bio mortar in comparison to the earth plain mortar. The EDS performed for the earth- and cement-based mortars shows that there is a decrease in the quantity of Si and K in the composition with the biostabiliser for both families of mortars (Table 3 and Table 4). However, that decrease is more impressive for the earth-based mortars with the biostabiliser, which might be associated with a dissolution in the silicate structures due to the presence of more water. This will be discussed more in the next topic on the adsorption and desorption of water vapour.

**Figure 13:**
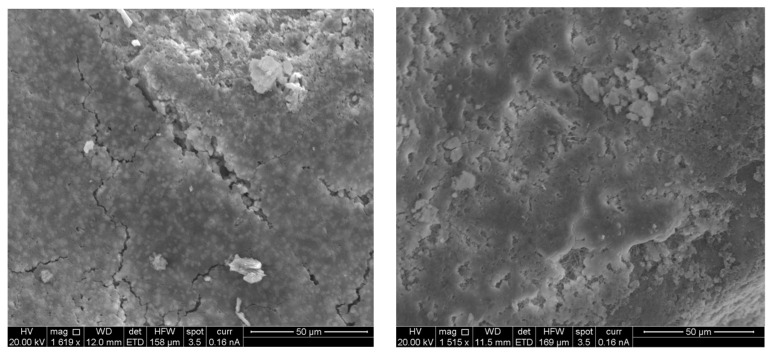
Cement plain mortar (**left**); cem+bio mortar (**right**).

**Figure 14:**
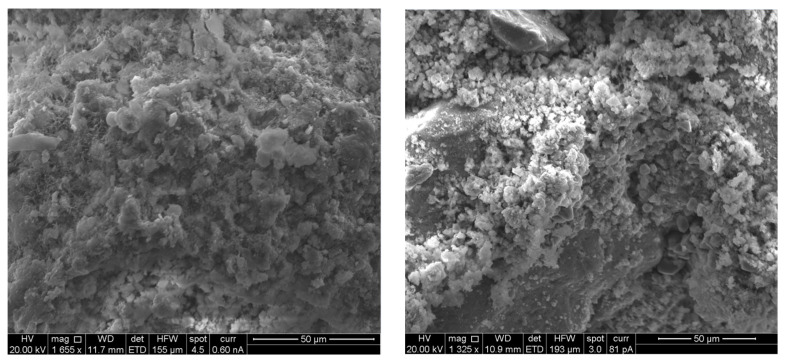
Earth plain mortar (**left**); earth+bio mortar (**right**).

A scanning electronic micrograph of cement and earth mortars with and without biostabiliser was completed after crushing for compressive strength at 28 days of age. The microstructure analysis of the samples is illustrated in Figure 13 (cement-based) and Figure 14 (earth-based). Figure 13 (left) demonstrates the SEM analysis of the cement plain sample with visible micro-cracks and voids. Figure 13 (right) shows the micro-structure of the cem+bio mortar, revealing the existence of crystalline calcium carbonate, which correlates with bacteria precipitation; nearly all the micro-cracks and voids are filled with calcium carbonate. Calcium carbonate in the form of calcite is detected not only in earth-based but also earth+bio mortars (Figure 14 (left and right) and Table 4). This increased quantity of calcium carbonate in the cement- and earth-based mortars with biostabiliser, as observed in the SEM of the cement and earth mortars and EDS, is the microstructure behaviour explanation of the macro behaviour detected for the mortars regarding the compressive and flexural tensile strength increase, as discussed below.

### 3.4. Adsorption and Desorption of Water Vapour

Adsorption is a surface process with the accumulation of water (or oxygen) on the solid mortar. Adsorption can be defined based on the strength of the interaction between the adsorbent (the substrate onto which chemicals attach) and the adsorbed molecules. The strength of the interaction can be due to Van der Waals interactions between the substrate and adsorbate (the molecule that is adsorbed, which, in this case, is water), or due to the chemical bonds involved (covalent bonds usually) in sticking the adsorbate to the adsorbent [34].

Desorption can occur when an equilibrium situation is altered and corresponds to the release of one substance from another, either from the surface or through the surface.

In this context, if the mortar porous media conditions favour the adsorption of a water molecule, then the molecule will adhere to the stationary phase and be separated from the other sample chemicals. When the conditions favour desorption, the opposite will occur and the chemicals will be released into the mobile phase. 

A combination of the NORDTEST protocol and ISO 21453 was employed in order to determine the moisture buffering value (MBV) of the mortars, which corresponds to the ability of the cement- and earth-based mortars to adsorb and desorb water vapour from the environment (Equation (1)) when the air humidity is high and low, respectively. The samples were covered with aluminium tape and laid horizontally with the single exposed surface pointing upwards. They were then exposed to 24 h cycles of RH of 75% for 8 h and 53% for 16 h at 23 °C Afterwards, they were exposed to a cyclical step change in RH of 75% for 8 h and 53% for 16 h in accordance with the test conditions defined by Romano et al. [35,36]. The results were obtained for cycles 1, 3, 8, and 10.

MBV = (ma − md)/(AΔφ)(1)

where

ma = Mass of mortar at end of moisture adsorption stage (g)
md = Mass of mortar at end of moisture desorption stage (g)
A = Exposed surface area of the mortar (m^2^)
Δφ = RH difference between adsorption and desorption stage (%)

**Figure 15:**
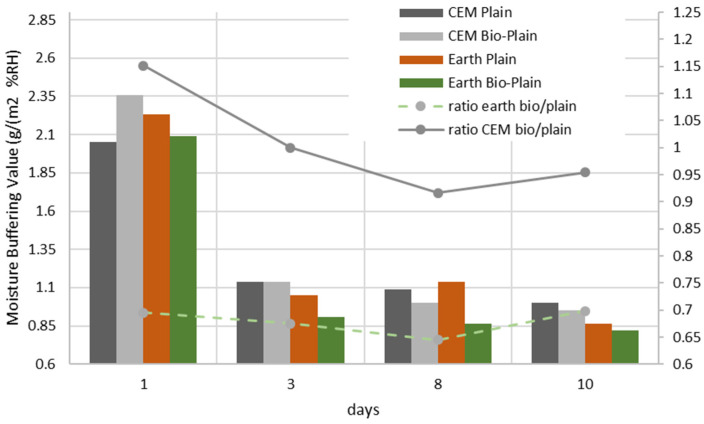
MBV for bio and non-bio earth and cement mortars after 1 month.

It is generally a desirable property for a material to have a high MBV [35,36]. Figure 15 shows that, for the earth mortars, the biostabiliser tends to lower the MBV in comparison to without the biostabiliser. At the 10th cycle, the results are similar in both situations. Probably, the precipitation decreases the ability of the earth-based mortar to absorb and desorb water vapour from the air, making the material more stable to relative humidity changes. The MBV for the cement mortars with the biostabiliser is at least 15% higher in the first day of cycles, but it tends to decrease with time, and, after three cycles, the MBVs are similar. For the earth mortars, the same biostabiliser can decrease the MBV by between 6 and 13%.

These changes in the MBV seem to happen from cycle three (3 days after the beginning of the test). According to Rode et al. [37], the tested mortars are classified as good when the MBV varies between 1 and 2 or has a value above 2, which is classified as ‘Excellent’.

**Figure 16:**
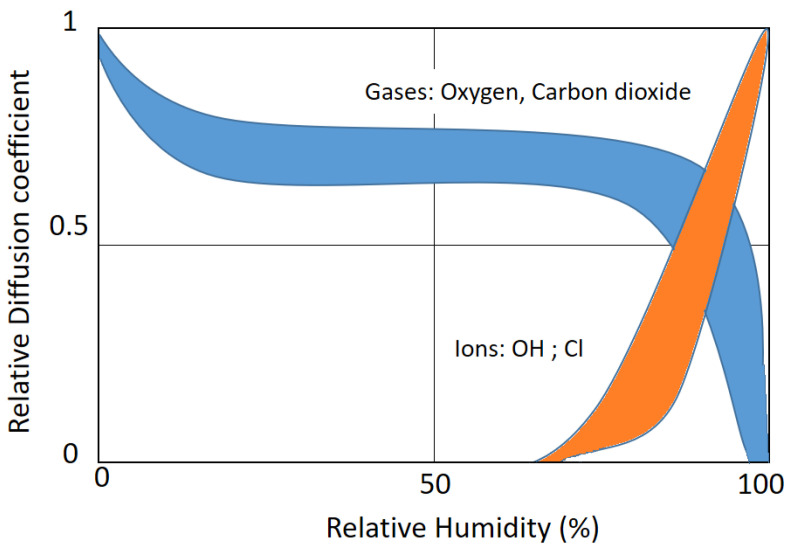
Schematic representation of the diffusion coefficients of ions and gases within a mortar as a function of the relative humidity based on Ref. [38].

With an increase in the RH cycle time, deeper layers of the mortars become involved in the diffusion process, which enables the transportation of water and ions due to the concentration gradient and partial pressure. It is the relative rates of adsorption and desorption onto and off of the stationary phase that allow the chemicals in the samples to be separated. At low relative humidity, capillary suction is more efficient, whereas, in wet/humid porous media, the diffusion coefficients decrease for gases but play a more important role for the mobility of ions, which is greatest in completely water-filled pores [21]. The sorption isotherm of the concrete influences the increase and decrease in the diffusion coefficient (Figure 16).

**Figure 17:**
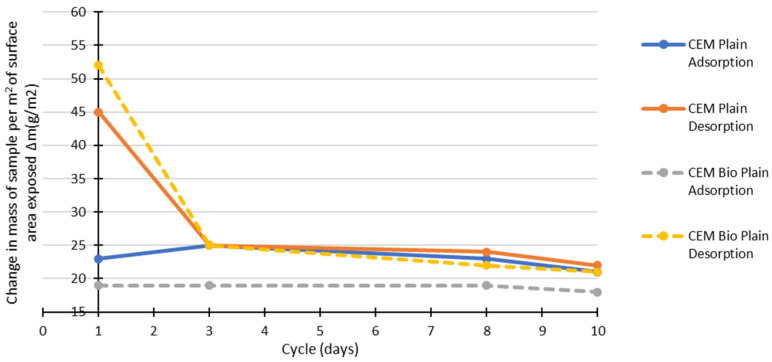
Adsorption and desorption change during 10 cycles for cement-based mortars with/without biostabiliser (bio).

**Figure 18:**
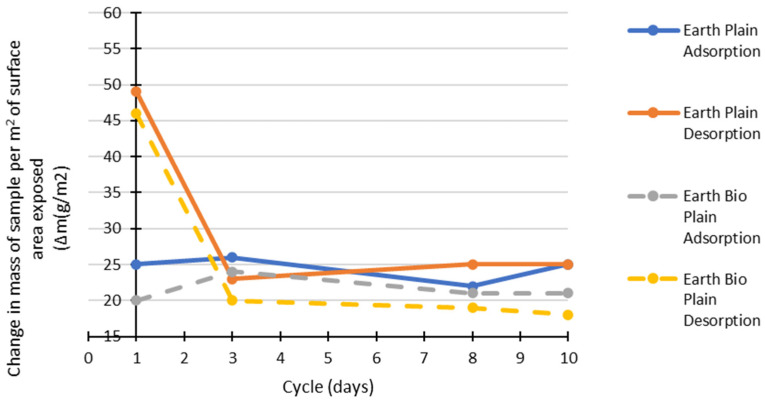
Adsorption and desorption change during 10 cycles for earth-based mortars with/without biostabiliser (bio).

The following figures (Figure 17 and Figure 18) show the contribution of the adsorption and desorption stages for cement- and earth-based mortars with or without the biostabiliser.

Adding the bioproduct leads to a decrease of water adsorption by 18% in the first 3 days of testing in comparison to the cement plain compositions (Figure 17). However, there is 15% more desorption in the first cycle for cem+bio in comparison to plain. After three cycles, there is more desorption than adsorption of water for cem+bio, but the plain compositions present similar absorption/desorption values. The results show that the biostabiliser effect through precipitation seems to decrease the ability of a cement-based mortar to adsorb more moisture from the air. Because the conditions favour desorption in cem+bio, the biostabiliser precipitation seems to facilitate the release of the chemicals into the mobile phase.

For the earth-based mortars (Figure 18), adding the bioproduct leads to a decrease of water adsorption by 20% in the first 3 days of testing in comparison to the earth plain compositions. However, there is 6% less desorption in the first cycle in comparison to plain and 20% less desorption in the following days if the bioproduct is added. After three cycles, there is less desorption than adsorption of water for the earth+bio, but earth plain seems to present a similar level of adsorption and desorption. The relative rates of adsorption and desorption onto and off of the stationary phase allow the chemicals in the samples to be separated. In this context, it seems that the precipitation in the earth+bio mortar porous media conditions favours the adsorption of water molecules, making the molecule adhere to the stationary phase and be separated from the other sample chemicals. The SEM/EDS performed for the earth- and cement-based mortars confirms the calcium carbonate precipitation and shows that there is a decrease in the quantity of Si and K in the composition with the biostabiliser for both families of mortars (Table 3 and Table 4). However, that decrease is more impressive for the earth-based mortars with the biostabiliser, which might be associated with a dissolution of silicate structures due to the presence of more water. This is in agreement with Ghosh et al. [39], who stated that *S. oneidensis* leaches silica. The work developed in Ref. [40] also confirms that the dissociation of the silicates occurs in processes that involve water molecule(s) (or the OH– group). The water molecule reduces the energy barrier to dissociate the Si–O–Si bridge. The dissociation is also favoured in the presence of the OH– groups, which increase if RH increases. This could help to understand how to control the dissociation of silicates and the formation of alkali silicates or aluminosilicates, such as zeolites and geopolymers. The results from this work could be extended to the chemistry around the formation of alkali silica gels during the so-called alkali–silica reaction, which can create irreversible damage in cement [41,42,43].

### 3.5. Compressive and Three-Point Flexural Strength

**Figure 19:**
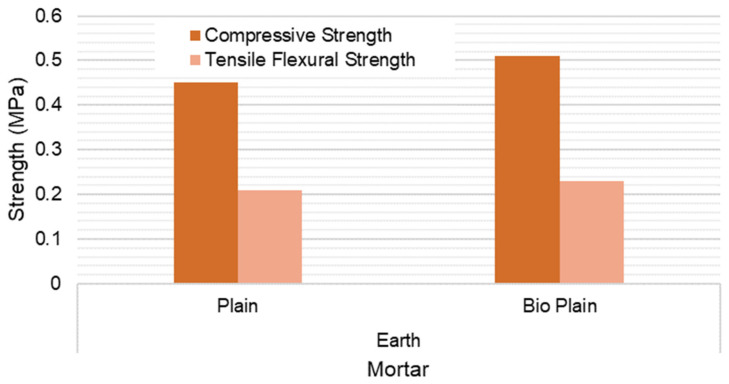
Compressive and tensile flexural strength at 28 days for the earth-based mortar with and without biostabiliser. Coefficient of variation for tensile flexural strength (CoV) per family of mortar: earth mortar: 23.4%; earth+bio: 14.0%. Coefficient of variation for compressive strength (CoV) per family of mortar: earth mortar: 16.6%; earth+bio: 11.2%.

**Figure 20:**
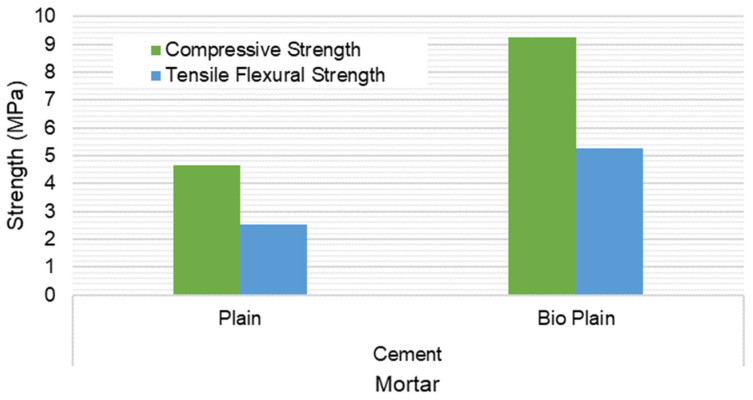
Compressive and tensile flexural strength at 28 days for the cement-based mortar with and without biostabiliser. Coefficient of variation for tensile flexural strength (CoV) per family of mortar: cement mortar: 1.3%; cem+bio: 4.9%. Coefficient of variation for compressive strength (CoV) per family of mortar: cement mortar: 4.3%; cem+bio: 3.6%.

The compressive and three-point flexural test results are presented in Figure 19 and Figure 20. The results show that there is a compressive and flexural strength increase for both compositions if the biostabiliser is used. However, the increase is significant mainly for the cement-based mortars. For the tested earth-based mortars, this increase is around 10% if the biostabiliser is added. This is in agreement with the SEM/EDS results obtained and discussed above. For the cement-based mortars (Figure 20), the strength increase with the biostabiliser is almost double that of the plain one, either for compressive or flexural strength.

Bacteria adhesion is the first stage for biofilm formation, where the bacteria–surface interactions are essential for biofilm control. Surface charge plays an important role in determining the binding force between bacteria and the surface, and it has long been known to affect biofilm formation. Most bacterial cells are negatively charged; thus, in general, a positively charged surface is more prone to bacterial adhesion, and a negatively charged surface is more resistant to bacterial adhesion [44,45]. In the case of earth-based mortar, the content in clay makes a surface with a negative charge due to its cation exchange capacity, which can explain the best relative performance of cem+bio in comparison to earth+bio. However, the clay surface can have its surface interaction changed by adding some dispersants from the cement or ceramic industry [46,47].

### 3.6. Open Porosity

**Figure 21:**
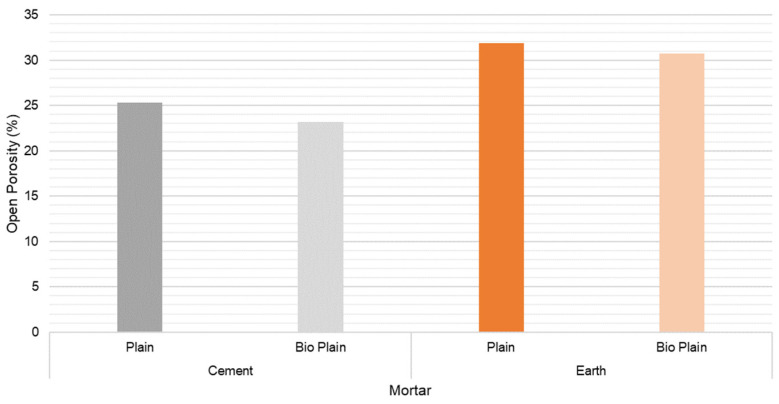
Open porosity at 28 days for the cement-based mortar with and without biostabiliser. Coefficient of variation (CoV) per family of mortar: cement mortar: 1.8%; cem+bio: 3.2%; earth mortar: 1.2%; earth+bio: 0.5%.

EN 1936:2006 was used to measure the open porosity of the mortars in order to quantify the void space inside the samples and understand the microstructure (Figure 21). Despite earth-based mortars presenting an open porosity of 30%, which is higher than the 25% for cement-based mortars, adding the biostabiliser only slighter decreases this value. However, the porosity results are in agreement with those of other studies focused on earth and cement [21,48].

## 4. Conclusions

This study investigated the potential of using mineral precipitation as a stabiliser in earthen construction materials. These biostabilisers have some advantages by providing suitable mechanical properties, durability performance, and improved hygroscopic behaviour in comparison to stabilisation with cement. Earth-based mortars’ performance with or without the biostabiliser was compared with cement-based mortars with or without the biostabiliser. Cultures of *S. oneidensis* with a concentration of 2.3 × 10^8^ cfu/mL were used. The biostabiliser was then used to prepare earth-based and cement-based mortars with a ratio of 6% of binder. The mechanical strength, water absorption via capillary, and moisture buffering results were analysed.

The capillary results indicate that this biostabiliser presents higher benefits to minimise the migration of water-soluble ions in the tidal-splash zones of earthen constructions than in underwater environments. It is observed that the initial water absorption tends to be lower for the samples with the biostabiliser than the ones without due to precipitation, as confirmed by micro and macro tests. The biostabiliser can prevent water migration more effectively for cement-based than for earth-based mortars in the initial stage of the capillary absorption. In the first 1 h of contact with water, the water absorption via the capillary tends to reduce by at least 60% for cem+bio, but, for earth+bio, it reduces by 5 to 10%. When the samples tend to become saturated, the biostabiliser shows a constant contribution of a 5% decrease in water absorption for the earth-based mortars. For the cement-based mortars, the contribution to decrease the absorption was above 15%. The long-term analysis shows, however, that a calcium carbonate precipitation effect is detected deeply in both families of mortars through the differences between the asymptotic value of the samples with or without the biostabiliser. This is confirmed through the SEM/EDS results, as discussed above.

At low relative humidity, the capillary suction is more efficient, whereas, in wet/humid concrete, the diffusion coefficients decrease for gases but play a more important role for the mobility of ions, which is greatest in completely water-filled pores. In an adsorption/desorption environment, the biostabiliser effect through precipitation seems to decrease the ability of a cement-based mortar to adsorb more moisture from the air. Because the conditions favour de-sorption in cem+bio, the biostabiliser precipitation seems to facilitate the release of the chemicals into the mobile phase. The precipitation in the earth+bio mortar porous media conditions favours the adsorption of water molecules, making the molecule adhere to the stationary phase and be separated from the other sample chemicals. The SEM/EDS performed for the earth- and cement-based mortars confirms the calcium carbonate precipitation and shows that there is a decrease in the quantity of Si and K in the composition with the biostabiliser for both families of mortars. However, that decrease is more impressive for the earth-based mortars with the biostabiliser, which might be associated with a dissolution of the silicate structures due to the presence of more water as S. *oneidensis* can leach silica. With an increase in the RH and the presence of OH– groups, the water molecules reduce the energy barrier to dissociate the Si–O–Si bridge. A future study should include longer exposure of samples to the humidity cycles in order to analyse the final MBV improvement of the mortars with the biostabilisers.

For the tested earth-based mortars, there was an increase of 10% for compressive and flexural strength if the biostabiliser was added. For the cement-based mortars, the strength increase was almost double that of the plain one. In the case of the earth-based mortars, the content in the clay makes a surface with a negative charge due to its cation exchange capacity, which can explain the best relative performance of cem+bio in comparison to earth+bio, meaning that the use of dispersants might improve the performance.

## Figures and Tables

**Table 1:** Earth-based and cement-based samples mix design (by weight).

Mortar	Lime	Earth	Sand
Earth-based	1	0.1	8.6
	**Cement**		**Sand**
Cement-based	1		3.7

**Table 2:** Testing procedures adopted in this study.

Tests Performed	Test in conformity with Standard	Picture
Bulk Density	EN 1015-6	
Compressive strength	EN 826 and EN 1015-11	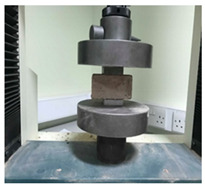
Three-point flexural strength	EN 12089	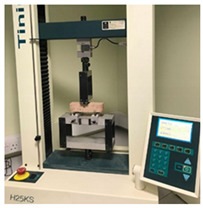
Porosity	EN 1936	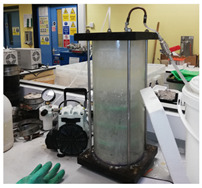
Water absorption via capillary	EN 1015-18 and EN 15801	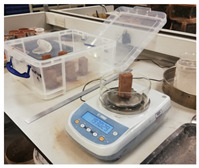
Scanning electron microscopy coupled energy-dispersive X-ray spectroscopy (SEM-EDS)	FEI Inspect S SEM variable vacuum. Kv range 0.1–30 kv used	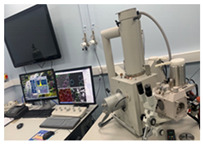
Moisture Buffering Volume	NORD TEST/ISO 21453	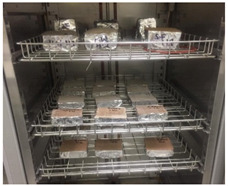

**Table 3:** EDS analysis of cement-based mortars with and without biostabiliser.

CaCO_3_	Ca		O		Si		K	
	Weight (%)	Atomic (%)	Weight (%)	Atomic (%)	Weight (%)	Atomic (%)	Weight (%)	Atomic (%)
Cement Plain Average	24.0	13.0	51.1	69.2	15.3	11.8	1.6	0.9
Cem+Bio Average	33.1	18.0	52.6	71.6	9.4	7.3	0.5	0.3

**Table 4:** EDS analysis of earth-based mortars with and without biostabiliser.

CaCO_3_	Ca		O		Si		K	
	Weight (%)	Atomic (%)	Weight (%)	Atomic (%)	Weight (%)	Atomic (%)	Weight (%)	Atomic (%)
Earth Plain Average	15.8	9.4	40.2	59.5	30.5	25.7	0.8	0.5
Earth+Bio Average	31.8	16.7	58.2	76.4	5.2	3.9	2.1	1.1

## Data Availability

The data presented in this study are available in cited references and on request from the corresponding author.

## References

[1] Kulshreshtha Y., Mota N., Jagadish K.S., Bredenoord J., Vardon P., van Loosdrecht M.C., Jonkers H. (2020). The potential and current status of earthen material for low-cost housing in rural India. Constr. Build. Mater..

[2] UK’s Third Climate Change Risk Assessment (CCRA3), UK Climate Change Risk Assessment 2021: Independent Assessment of UK Climate Risk 2021. https://www.theccc.org.uk/publication/independent-assessment-of-uk-climate-risk/.

[3] Van den Berg R.D., Naidoo I., Tamondong S.D. (2017). Evaluation for Agenda 2030: Providing Evidence on Progress and Sustainability.

[4] Losini A., Grillet A., Bellotto M., Woloszyn M., Dotelli G. (2021). Natural additives and biopolymers for raw earth construction stabilization—A review. Constr. Build. Mater..

[5] Morel J.-C., Charef R., Hamard E., Fabbri A., Beckett C., Bui Q.-B. (2021). Earth as construction material in the circular economy context: Practitioner perspectives on barriers to overcome. Philos. Trans. R. Soc. B Biol. Sci..

[6] Fabbri A., Aubert J.-E., Bras A.A., Faria P., Gallipoli D., Goffart J., McGregor F., Perlot-Bascoules C., Soudani L., Fabbri A., Morel J.C., Aubert J.E., Bui Q.B., Gallipoli D., Reddy B.V. (2022). Hygrothermal and Acoustic Assessment of Earthen Materials. Testing and Characterisation of Earth-Based Building Materials and Elements.

[7] Fabbri A., Morel J.-C., Gallipoli D. (2018). Assessing the performance of earth building materials: A review of recent developments. RILEM Tech. Lett..

[8] Faria P., Santos T., Aubert J.-E. (2016). Experimental Characterization of an Earth Eco-Efficient Plastering Mortar. J. Mater. Civ. Eng..

[9] Hall M., Allinson D. (2009). Analysis of the hygrothermal functional properties of stabilised rammed earth materials. Build. Environ..

[10] Hall M., Allinson D. (2009). Assessing the effects of soil grading on the moisture content-dependent thermal conductivity of stabilised rammed earth materials. Appl. Therm. Eng..

[11] Laborel-Préneron A., Aubert J.E., Magniont C., Tribout C., Bertron A. (2016). Plant aggregates and fibers in earth con-struction materials: A review. Constr. Build. Mater..

[12] Mcgregor F., Heath A., Fodde E., Shea A. (2014). Conditions affecting the moisture buffering measurement performed on compressed earth blocks. Build. Environ..

[13] McGregor F., Heath A., Maskell D., Fabbri A., Morel J.-C. (2016). A review on the buffering capacity of earth building materials. Proc. Inst. Civ. Eng.-Constr. Mater..

[14] Melià P., Ruggieri G., Sabbadini S., Dotelli G. (2014). Environmental impacts of natural and conventional building materials: A case study on earth plasters. J. Clean. Prod..

[15] Sujatha E.R., Saisree S. (2019). Geotechnical behaviour of guar gum-treated soil. Soils Found..

[16] Bras A.A., Addo I.A., Beckett C.T., Yakubu I., Owusu-Nimo F., Gagnon A., Arora M., Huang Y. Biological methods to increase housing resilience to flooding. Proceedings of the World Resources Forum Conference.

[17] Canakci H., Sidik W., Kilic I.H. (2015). Effect of bacterial calcium carbonate precipitation on compressibility and shear strength of organic soil. Soils Found..

[18] Ivanov V., Chu J. (2008). Applications of microorganisms to geotechnical engineering for bioclogging and biocementation of soil in situ. Rev. Environ. Sci. Bio/Technol..

[19] Van der Star W., Rossum W.V.W.-V., van Paassen L., van Baalen L., van Zwieten G. Stabilization of gravel deposits using microorganisms. Proceedings of the 15th European Conference on Soil Mechanics and Geotechnical Engineering.

[20] Martinez B.C., DeJong J.T., Ginn T.R., Montoya B.M., Barkouki T.H., Hunt C., Tanyu B., Major D. (2013). Experimental Optimization of Microbial-Induced Carbonate Precipitation for Soil Improvement. J. Geotech. Geoenviron. Eng..

[21] Romano A., Mohammed H., de Sande V.T., Bras A. (2021). Sustainable bio-based earth mortar with self-healing capacity. Proc. Inst. Civ. Eng.-Constr. Mater..

[22] Tayebani B., Mostofinejad D. (2019). Self-healing bacterial mortar with improved chloride permeability and electrical resistance. Constr. Build. Mater..

[23] Tittelboom K., Belie N. (2013). Self-Healing in Cementitious Materials—A Review. Materials.

[24] Ammari A., Bouassria K., Zakham N., Cherraj M., Bouabid H., D’ouazzane S. (2018). Durability of the earth mortar: Physico-chemical and mineralogical characterization for the reduction of the capillary rise. MATEC Web Conf..

[25] Chabriac P.-A., Morel J.-C., Fabbri A., Blanc-Gonnet J., Hans S. A case study of the hygrothermal behaviour of rammed earth building. Proceedings of the 8th Conference on Sustainable Development of Energy, Water and Environment Systems.

[26] Eid J. (2018). New construction material based on raw earth: Cracking mechanisms, corrosion phenomena and physico-chemical interactions. Eur. J. Environ. Civ. Eng..

[27] Chou C.-W., Seagren E.A., Aydilek A.H., Lai M. (2011). Biocalcification of Sand through Ureolysis. J. Geotech. Geoenviron. Eng..

[28] Ma W., Han Y., Ma W., Han H., Zhu H., Xu C., Li K., Wang D. (2017). Enhanced nitrogen removal from coal gasification wastewater by simultaneous nitrification and denitrification (SND) in an oxygen-limited aeration sequencing batch biofilm reactor. Bioresour. Technol..

[29] Choi S.-G., Chang I., Lee M., Lee J.-H., Han J.-T., Kwon T.-H. (2020). Review on geotechnical engineering properties of sands treated by microbially induced calcium carbonate precipitation (MICP) and biopolymers. Constr. Build. Mater..

[30] Sharma G., Sharma S., Kumar A., Al-Muhtaseb A.H., Naushad M., Ghfar A.A., Mola G.T., Stadler F.J. (2018). Guar gum and its composites as potential materials for diverse applications: A review. Carbohydr. Polym..

[31] Larson S.L., Newman J.K., Griggs C.S., Beverly M., Nestler C.C. (2012). Biopolymers as an Alternative to Petroleum-Based Polymers for Soil Modification.

[32] Nakamatsu J., Kim S., Ayarza J., Ramírez E., Elgegren M., Aguilar R. (2017). Eco-friendly modification of earthen construction with carrageenan: Water durability and mechanical assessment. Constr. Build. Mater..

[33] Mohammed H., Giuntini F., Sadique M., Shaw A., Bras A. (2021). Polymer modified concrete impact on the durability of infrastructure exposed to chloride environments. Constr. Build. Mater..

[34] Chromatrography Today. https://www.chromatographytoday.com/news/hplc-uhplc/31/breaking-news/adsorption-absorption-and-desorption-whats-the-difference/31397#.Yi8sacWJAdE.link.

[35] Romano A., Bras A., Grammatikos S., Shaw A., Riley M. (2019). Dynamic behaviour of bio-based and recycled materials for indoor environmental comfort. Constr. Build. Mater..

[36] Romano A., Grammatikos S., Riley M., Bras A. (2020). Physicochemical characterisation of bio-based insulation to explain their hygrothermal behaviour. Constr. Build. Mater..

[37] Rode C., Peuhkuri R.H., Hansen K.K., Time B., Svennberg K., Arfvidsson J., Ojanen T. (2005). NORDTEST Project on Moisture Buffer Value of Materials. AIVC 26th Conference: Ventilation in Relation to the Energy Performance of Buildings. Air Infiltration and Ventilation.

[38] Hunkeler F. (2005). Corrosion in reinforced concrete: Processes and mechanisms. Corros. Reinf. Concr. Struct..

[39] Ghosh S., Biswas M., Chattopadhyay B., Mandal S. (2009). Microbial activity on the microstructure of bacteria modified mortar. Cem. Concr. Compos..

[40] Dupuis R., Pellenq R.J.-M., Champenois J.-B., Poulesquen A. (2020). Dissociation Mechanisms of Dissolved Alkali Silicates in Sodium Hydroxide. J. Phys. Chem. C.

[41] Gavali H.R., Bras A., Faria P., Ralegaonkar R. (2019). Development of sustainable alkali-activated bricks using industrial wastes. Constr. Build. Mater..

[42] Benmore C., Monteiro P.J. (2010). The structure of alkali silicate gel by total scattering methods. Cem. Concr. Res..

[43] Kirkpatrick R.J., Kalinichev A.G., Hou X., Struble L. (2005). Experimental and molecular dynamics modeling studies of interlayer swelling: Water incorporation in kanemite and ASR gel. Mater. Struct..

[44] Song F., Koo H., Ren D. (2015). Effects of Material Properties on Bacterial Adhesion and Biofilm Formation. J. Dent. Res..

[45] Teughels W., Van Assche N., Sliepen I., Quirynen M. (2006). Effect of material characteristics and/or surface topography on biofilm development. Clin. Oral Implant. Res..

[46] Cornell University Agronomy Fact Sheet #22: Cation Exchange Capacity (CEC). http://nmsp.cals.cornell.edu/publications/factsheets/factsheet22.pdf.

[47] Landrou G., Brumaud C., Habert G. (2017). Clay particles as binder for earth buildings materials: A fresh look into rheology of dense clay suspensions. EPJ Web Conf..

[48] Van der Bergh J.M., Miljević B., Šovljanski O., Vučetić S., Markov S., Ranogajec J., Bras A. (2020). Preliminary approach to bio-based surface healing of structural repair cement mortars. Constr. Build. Mater..

